# Brain network mechanism of acupuncture for chronic spontaneous urticaria: a functional magnetic resonance imaging study protocol

**DOI:** 10.3389/fneur.2023.1263753

**Published:** 2023-10-03

**Authors:** Xianhao Huang, Jing Xie, Yang Yang, Xuechun Dai, Lingyun Lu, Ning Li, Ying Li, Song Wang, Leixiao Zhang

**Affiliations:** ^1^Division of Internal Medicine, Institute of Integrated Traditional Chinese and Western Medicine, West China Hospital, Sichuan University, Chengdu, China; ^2^Chengdu Xinjin District Hospital of Traditional Chinese Medicine, Chengdu, China; ^3^Acupuncture and Tuina School, Chengdu University of Traditional Chinese Medicine, Chengdu, China; ^4^Functional and Molecular Imaging Key Laboratory of Sichuan Province, Department of Radiology and Huaxi MR Research Center (HMRRC), West China Hospital, Sichuan University, Chengdu, China

**Keywords:** acupuncture, chronic spontaneous urticarial, microbiota, fMRI, randomized controlled trial, protocol

## Abstract

**Introduction:**

Chronic spontaneous urticaria (CSU) is a common skin condition that can significantly impact patients’ quality of life. Although studies have demonstrated the efficacy of acupuncture in treating CSU, the underlying mechanisms remain unclear. Dysfunction within the brain’s default mode network (DMN) represents a fundamental characteristic of central pathological changes associated with CSU. Therefore, it is hypothesized that improving brain network dysfunction could serve as a key mechanism through which acupuncture exerts its therapeutic effects. This study aims to provide evidence supporting this hypothesis.

**Methods and analysis:**

This study, a parallel, randomized, sham-controlled functional neuroimaging investigation will be conducted in China. We aim to enroll 50 patients with CSU and 25 healthy controls, distributing them evenly between the acupuncture and sham acupuncture groups in a 1:1 ratio. The total observation period will span 6 weeks, including 2 weeks designated for the baseline phase and 4 weeks allocated for the clinical treatment phase. Prior to treatment, all participants will undergo magnetic resonance scanning, clinical index detection, and microbiota collection. Following treatment, the patients with CSU will be retested for these indicators. Using resting-state functional connectivity (rsFC) analysis, dynamic Functional Connection (dFC) analysis, and brain microstate extraction technology combined with correlation analysis of microbiota and clinical indicators, the regulatory mechanism of acupuncture on the brain network of CSU will be evaluated from multiple dimensions.

**Ethics and dissemination:**

This trial was approved by the Biomedical Ethics Review Committee of the West China Hospital, Sichuan University (No. 2022-1255). Each participant will provide written informed consent to publish any potentially identifiable images or data.

**Clinical trial registration**https://www.chictr.org.cn/, identifier: ChiCTR2200064563.

## Introduction

Chronic urticaria (CU) is a skin disease characterized by reddish wheals and unbearable itching, with or without angioedema, lasting for ≥6 weeks. Chronic spontaneous urticaria (CSU) is the most common type of CU (71.2%) and is characterized by urticaria symptoms without inducing factors (1, 2). The prevalence of CU is particularly high within the Asian population (1.5%), with a higher prevalence among women than men (3). Owing to complicated conditions, recurrent symptoms, and few effective curative treatments, patients are often required to take high-cost drugs for an extended period. This significantly impacts their quality of life and places a considerable burden on both individuals and society (4, 5).

The latest clinical guidelines for urticaria state that modern second-generation H1-antihistamines should be employed as first-line treatment drugs (2). Despite this, the guidelines do not provide specific recommendations for individual specific drugs due to insufficient research on the effectiveness and safety evaluation of all second-generation H1-antihistamines. Furthermore, the guidelines recommend using omalizumab or cyclosporine for patients who have an inadequate response to second-generation H1-antihistamines. However, there are still some patients who do not benefit from these medications (6, 7). The Taiwanese Dermatological Association consensus proposes that non-pharmacological therapies are also an important approach in alleviating urticaria (8). As an nondrug alternative therapy, acupuncture has been widely used to treat pruritic dermatoses, including CSU, and has demonstrated curative effects (9, 10). However, due to a lack of high-quality evidence supporting its use and insufficient elucidation on the mechanisms underlying acupuncture’s effects, recent clinical practice guidelines do not recommend or report the beneficial effect of acupuncture on CSU (11, 12). Therefore, further research is needed to provide additional evidence.

Given the multi-target and holistic regulatory characteristics of acupuncture, single-mechanism studies cannot fully elucidate the mechanism underlying its therapeutic effects. Therefore, the objective of this study is to explore the potential therapeutic targets of acupuncture by examining a systematic onset mechanism on CSU. Functional magnetic resonance imaging (fMRI) studies have demonstrated that during episodes of dermatotic itch in patients, the primary afferent nerves from the skin projects itch signals to the thalamus. These signals activate the brain’s default mode network (DMN) (13, 14), leading to abnormal functional connectivity. It has been established that DMN brain dysfunction represents an important characteristic of central pathological changes in CSU (15, 16). Moreover, Characteristic changes have been reported in the intestinal microbial population of patients with CSU (17), which may aggravate inflammatory responses and immune dysfunction (18). Similar to gut microbiota, the skin microbiota is closely associated with chronic inflammatory skin diseases (19–21). It plays a regulatory role in modulating immune responses within the skin to prevent inflammation (22). Furthermore, a bidirectional interaction between the microbiota and brain function, often referred to as the “Microbiota-Brain Axis,” has been identified (23). This interaction may potentially contribute to the pathogenesis of certain chronic inflammatory skin conditions like rosacea (24), and can influence neurophysiology and behavior, including anxiety and depression. These effects involve complex mechanisms involving the immune system, neuroendocrine system, and metabolism (25). However, a comprehensive understanding of these mechanisms remain elusive. Another objective of this study is to enhance the understanding of the underlying pathological mechanisms involved in CSU from diverse perspectives.

Recent studies on brain networks have shown that DMN largely overlaps with acupuncture response areas (26, 27). That is, verum acupuncture can regulate the DMN and increase the functional connectivity of the DMN with the sensorimotor network, pain emotion, and memory-related brain regions. Moreover, acupuncture can exert therapeutic effects by modulating microbiota (28, 29). These underlying mechanisms also play a crucial role in both the development and resolution of CSU. Accordingly, we have designed a randomized sham acupuncture-controlled trial. fMRI will serve as the primary research method while the key DMN brain regions regulated by acupuncture for CSU treatment will represent the seed points. Furthermore, we plan to analyze the microbiota and clinical indicators. The primary objective of this study is thus to systematically elucidate the brain network mechanism associated with the treatment of CSU by acupuncture. Our goal is to provide robust scientific evidence to support the clinical application of acupuncture as a treatment modality for CSU.

## Methods and analysis

### Study design

**Figure 1:**
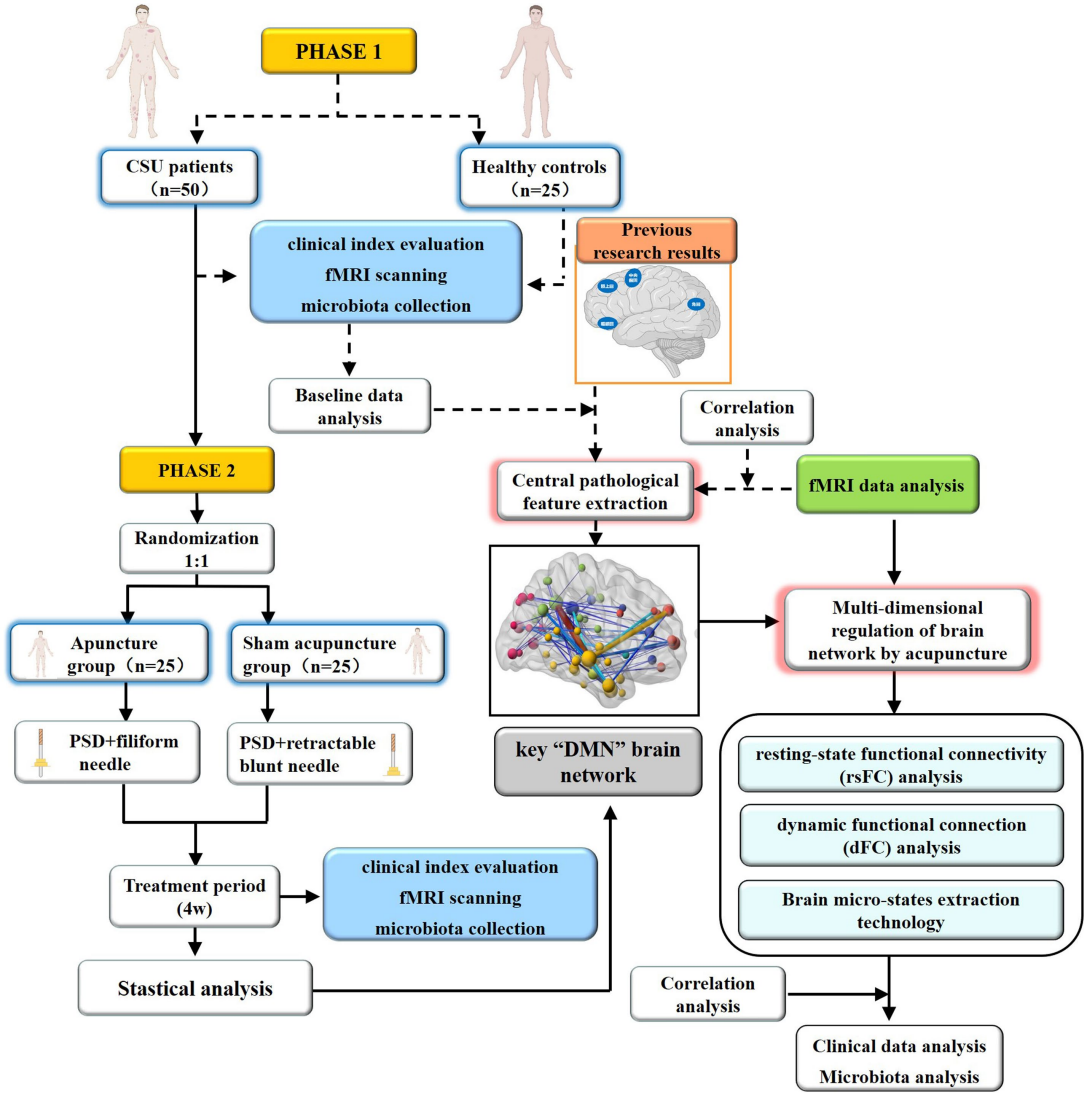
Flow diagram.

This is a parallel randomized sham-controlled functional neuroimaging study. Fifty CSU patients and 25 matched healthy subjects will be randomly assigned to the verum or sham acupuncture groups at a 1:1 ratio. The total observation period of this study is 6 weeks, including a baseline period of 2 weeks and a clinical treatment period of 4 weeks. Before treatment, all participants (*n* = 75) will undergo clinical index evaluation, magnetic resonance scanning, and microflora collection. Following treatment, only the CSU patients will be reassessed for these indicators. The study flow chart is presented in Figure 1, and the schedule of enrolment, interventions, and assessments is shown in Table 1. This trial will be reported following the Standard Protocol Items: Recommendations for Interventional Trials (SPIRIT).

**Table 1:** Schedule of enrolment, interventions, and assessments.

Time point	Enrolment	Baseline	Treatment period	
	Week-1	Week 0	Week 2	Week 4
**Enrolment**				
Eligibility screen	√			
Informed consent	√			
Inclusion/exclusion criteria	√			
Expectation of acupuncture	√			
Physical examination	√	√	√	√
Medication record	√	√	√	√
Treatment record	√	√	√	√
**Intervention groups**				
Verum acupuncture			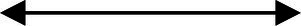	
Sham acupuncture				
**Assessments**				
UAS7/VAS		√	√	√
PSQI/DLQI		√	√	√
HAMA/HAMD		√	√	√
SAS/SDS		√	√	√
Skin wheal measures		√	√	√
IgE/CRP		√		√
fMRI		√		√
Gut/skin microbiota		√		√
**Safety**				
Safety evaluation			√	√
Adverse events				√
Effects self-assessment				√
Patient compliance				√
Blinding assessment				√

### Recruitment and informed consent

Participants will be recruited from the West China Hospital of Sichuan University from 1 January 2022 to 31 December 2023. Different strategies will be used to recruit potential participants, including posters and social media advertisements. Researchers will ensure that the participants are fully informed regarding the research procedures, benefits, and potential risks. Those who agree to participate will provide written informed consent before beginning the study and will be made aware that they can withdraw at any time.

### Participants

#### CSU patients

##### Diagnosis criteria

According to the diagnostic criteria of CSU in “the International EAACI/GA^2^LEN/EuroGuiDerm/APAAACI guidelines for the definition, classification, diagnosis, and management of urticaria” (2021 edition) (2): (1) repeat appearance of wheals, (2) with/without angioedema, (3) wheal episodes ≥2 times per week, (4) recurrent attacks ≥6 weeks, and (5) spontaneous wheals induced by nonspecific stimulation factors.

##### Inclusion criteria

The study will enroll patients who meet the following criteria (1) meet the diagnostic criteria of CSU as defined in “The international EAACI/GA^2^LEN/EuroGuiDerm/APAAACI guideline for the definition, classification, diagnosis, and management of urticaria” (2); (2) right-handedness of either sex, aged between 18 and 70 years, with a minimum of 6 years of education; (3) urticaria activity score 7 (UAS7) > 14; (4) no metal implants, no fMRI scanning contraindications; (5) patients who have not taken any antihistamines within 2 weeks before entering the study, and have not used steroid hormones or immunosuppressants within the last month; (6) no acupuncture treatment within 3 months before entering the study, no participation in other ongoing clinical studies; (7) and patient who have provided signed informed consent and volunteered to participate in this study.

##### Exclusion criteria

Patients who meet any of the following criteria will be excluded: (1) claustrophobic or other fMRI scanning contraindications; (2) unable to understand or record in the urticaria diary; (3) pregnant or lactating; (4) severe primary diseases, such as cardiovascular, liver, kidney, digestive, or hematopoietic conditions; (5) progressive malignant tumors or other severe wasting diseases that increase the risk of concurrent infection and bleeding; (6) unconscious, unable to express subjective symptoms independently, or diagnosed with a psychiatric illness; (7) and patients who have participated in similar research within the past month.

#### Healthy controls

##### Inclusion criteria

Participants who meet all of the following criteria will be included in this study: (1) have not participated in similar research within 1 month; (2) right-handedness of either sex; between the ages of 18 and 70 years, with a minimum of 6 years of education; (3) no history of urticaria or other allergic diseases; (4) no drug use for at least 15 days before entering the study; (5) no metal implants, no fMRI scanning contraindications; (6) all physiological indexes are common, no functional or organic diseases; (7) No participation in similar or other clinical studies; (8) volunteer for this study and provide signed informed consent.

##### Exclusion criteria

Participants who meet any of the following criteria will be excluded: (1) CSU disease history or allergic constitution; (2) pregnant or lactating; (3) Hamilton anxiety scale score (HAMA) > 7 or Hamilton depression scale score (HAMD) > 7; (4) metal implants, fMRI examination contraindications like claustrophobia; (5) severe asymmetry of skull anatomical structure or apparent lesions detected while scanning; (6) unconscious or unable to express subjective symptoms independently.

### Randomization and blinding

Eligible patients will be randomly assigned to the acupuncture or sham acupuncture group at a 1:1 ratio. Statistical analysis will be performed using SPSS software (version 22.0) to generate random sequences with a random number table. The random distribution cards will contain random numbers, serial numbers, and groups, packed in opaque numbered envelopes. The envelope will be unsealed following study enrolment. Owing to the particularity of acupuncture research, it is difficult to blind the acupuncture operators. As such, fake acupuncture devices and separate treatment rooms will be used for each patient to blind them as much as possible. Moreover, the “three separations strategy” will be strictly adhered to by researchers, operators, and statisticians throughout the research process. Efficacy evaluation, data analysis, and statistics will be completed by third parties blinded to the groupings.

### Interventions

Patients in the acupuncture and sham acupuncture groups will receive treatment for 4 weeks, five times per week for the first 2 weeks, and three times per week for the next 2 weeks, for a total of 16 treatments. The acupuncture group will be treated with filiform needles (Huatuo brand, 0.25 × 40 mm), whereas the sham group will be treated with retractable blunt needles (AcuPrime brand, 0.25 × 40 mm). A Park sham device (PSD) (DONGBANG Acupuncture Inc.) will be used to achieve patient blinding in both groups. Acupuncturists with a doctor’s qualification certificate and more than 5 years of experience will complete the treatment sessions. Before the formal start of the study, we will conduct a unified training and assessment of related acupuncture operations.

#### Acupuncture group

**Figure 2:**
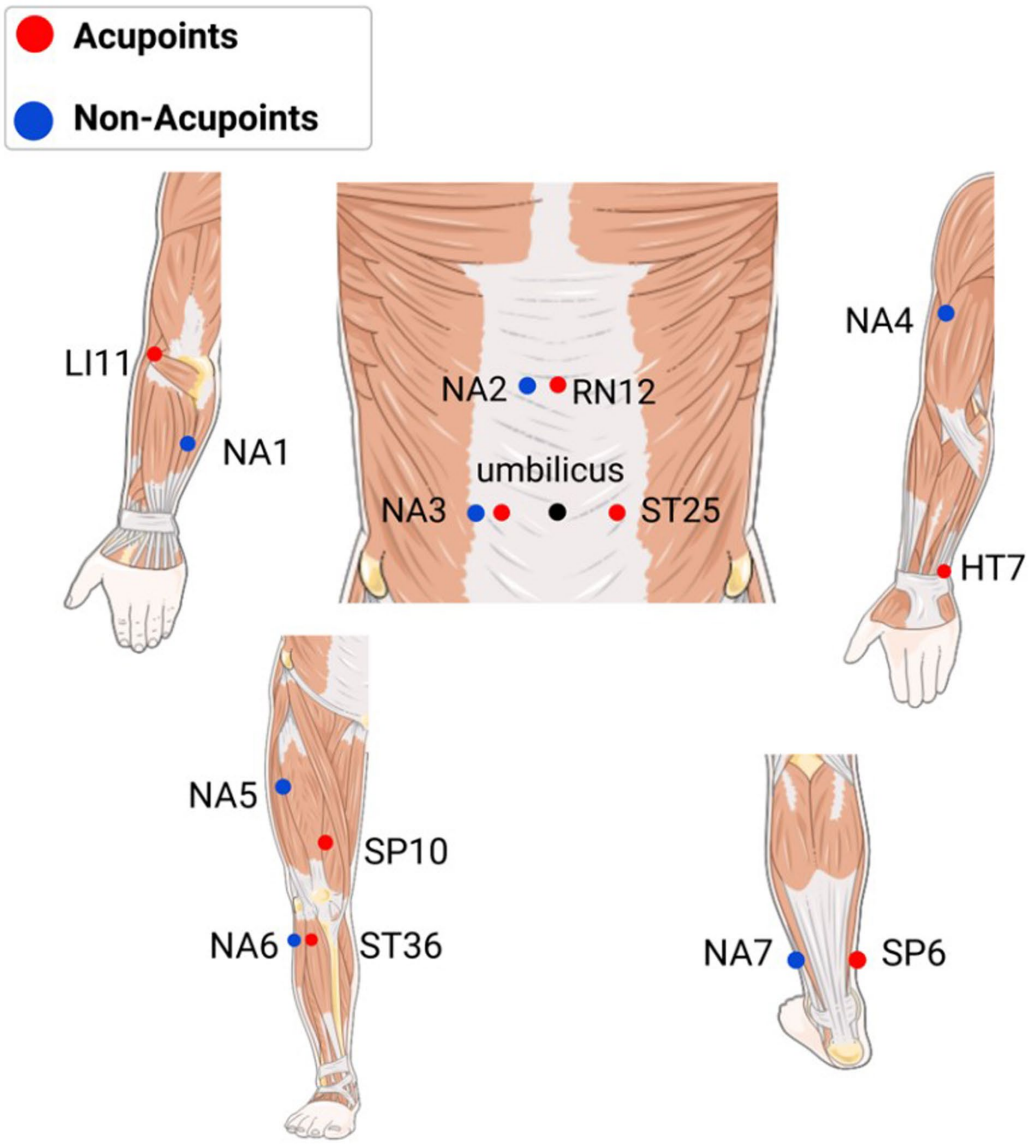
Location of acupoints and non-acupoints. NA1: midpoint between the medial epicondyle of the humerus and the ulnar malleolus, on the ulnar margin. NA2: 1.2 cun beside CV12. NA3: 1 cun lateral to ST25, the midpoint of ST25 and SP15. NA4: inner anterior edge of the arm, at the junction of the deltoid and biceps. NA5: on the thigh, 0.8 cun medial to the midpoint of the line connecting the anterior superior iliac spine and the outer superior angle of basis patellae. NA6: 1 cun lateral to ST36, between the stomach and gallbladder meridian. NA7: 3 cun above the prominence of the lateral malleolus, between the stomach and gallbladder meridian.

The acupuncture group will be treated with “PSD + filiform needle” combined equipment. The acupoint selection plan refers to the Chinese textbook *Acupuncture and moxibustion Science* and expert opinions (30). Bilateral acupoints: LI11 (Quchi), SP10 (Xuehai), SP6 (Sanyinjiao), ST36 (Zusanli), ST25 (Tian Shu), and HT7 (Shenmen); single acupoint: CV12 (Zhongwan). The acupoint locations are shown in Figure 2. The patients will be placed in the supine position. The operator will remove the release paper from the PSD surface and insert the needle through the device with the needle tip exposed at the end. After disinfecting the acupoints and inserting the needles, the PSD will be fixed. After inserting the needle (20–40 mm, vertically), the lifting-thrusting method will be applied to induce and maintain the Deqi sensation (frequency: 60–90 times/min, amplitude: 3–5 mm). The needles will be retained for 30 min.

#### Sham acupuncture group

The sham group will be treated with the “PSD + retractable blunt needle” combined equipment. Non-meridian and non-acupoint points will be selected (Figure 2). The retractable blunt needle has a hollow handle, and the needle tip is flat and blunt. Upon insertion, the body of the needle retracts into the hollow needle handle and does not penetrate the acupoints, remaining on the skin surface without causing deqi sensations. The needle will be retained for 30 min without needle manipulation. The remaining operating procedures will be performed in the same manner as that described for the acupuncture group.

### Outcome measures

#### Primary outcome measure

##### Urticaria activity score 7

The UAS7—a fundamental scale for evaluating urticaria symptoms (2)—assesses the number of wheals and degree of itching. Owing to the frequent and varying attacks of urticaria, we recommend daily recording. Subsequently, a total score of seven consecutive days will be used to evaluate urticaria activity.

#### Secondary outcome measures

##### Visual analog scale

The VAS will be used to evaluate the degree of urticarial itching in subjects over 1 week. More specifically, the participants will use a 10 cm horizontal line marked from 0 (no itching) to 10 (intolerable itching) to indicate their itching sensation. The degree of itching will be evaluated by measuring the distance from 0 to the mark. As CSU attacks occur more than once a day, the record is based on the highest daily VAS itching score.

##### Dermatology life quality index

The DLQI will be used to evaluate quality of life. This index covers six areas: symptoms and feelings, daily activities, leisure, work and school, interpersonal relationships, and the impact of treatment on everyday life (31).

##### Pittsburgh sleep quality index

The PSQI comprises nine questions used to evaluate the effect of CSU on sleep quality. The evaluation includes sleep quality, sleep latency, sleep duration, sleep efficiency, sleep disorders, sleeping pills, and daytime dysfunction (32).

##### Hamilton anxiety/depression scale

The Hamilton Anxiety Scale (HAMA) consists of 14 items (33), whereas the Hamilton Depression Scale (HAMD)_comprises 17 items (34). Both of these scales will be employed to evaluate the impact of CSU on the mental health of patients.

##### Self-rating anxiety/depression scale

The Self-rating anxiety scale (SAS) (35) and Self-rating depression scale (SDS) (36) are both self-report questionnaires consisting of 20 items each, designed to assess levels of anxiety and depression. Both have been widely used in mental health assessments among patients with various skin and other medical conditions (37).

#### Skin wheal measures

Infrared thermometers (Cofoe; Cofoe Medical Technology Co., LTD) will be used to measure the skin temperature of wheals. We will also use skin analyzers (RealBubee, Ningbo Realbubee Medical Equipment Co., LTD) to measure skin moisture, oil, and elasticity, as well as Vernier calipers to measure each wheal’s maximum length and vertical diameter. The arithmetic mean of the measured values will be recorded as the typical value.

### Serum laboratory testing

Patients with CSU exhibit higher levels of serum-specific IgE and C-reactive protein (CRP) compared to healthy controls (38, 39). Therefore, CSU patients will receive fasting hemospasia before and after treatment to detect serum-specific IgE and CRP levels.

### Gut microbiota collection

Stool samplers will be used to collect specimens from the middle of the stool (approximately 3 g) at the hospital. Samples will then be placed in a 10 mL centrifuge tube and immediately stored at −80°C. The genomic DNA of samples will be extracted by cetyltrimethylammonium bromide (CTAB) or sodium dodecyl sulfate (SDS) methods, while agarose gel electrophoresis will be employed to assess the purity and concentration of the extracted DNA. Subsequently, DNA will be collected in a centrifuge tube and diluted with sterile water to 1 ng/μL for further analysis.

### Skin microbiota collection

A sterile cotton swab soaked with normal saline will be applied to the skin surface (patients: skin at the wheals; healthy persons: corresponding normal skin). The sampling surface will be wiped smoothly in the horizontal and vertical directions, respectively. Subsequently, the bottom of the cotton swab head will be cut off at three sampling points, placed in a 15 mL centrifuge tube, and stored at −80°C. The DNA extraction and detection methods were the same as those for gut microbiota collection.

### fMRI outcome measures

We will use a Siemens’ 3.0 T superconducting magnetic resonance scanner and a 12-channel head coil for magnetic resonance scanning. All subjects will be scanned with the same parameters, and the same operator will be responsible for routine operations. An operator instruction manual will be produced to standardize the operation process and communication with the subjects. The various factors that can impact the magnetic resonance scanning (MRS) process will be strictly regulated. That is, the scanning time will be unified and the interference of the environment, equipment, technicians, and psychology on the subjects’ brain function imaging data will be limited, improving data reliability. The scanning parameters will be as follows: repetition time (TR): 2,000 ms, echo time (TE): 30 ms, field of view (FOV): 240 × 240 mm^2^, slice thickness (ST): 4 mm, number of slices (NOS): 40, matrix: 64 × 64, flip angle (FA): 90°.

### Safety assessment

If adverse events, such as dizziness, pain, bleeding, or haematoma occur after acupuncture, researchers will deal with them in a timely manner by inviting expert to consult when necessary to ensure the safety of the participants. The event’s occurrence time, symptoms, signs, severity, duration, and treatment methods will be recorded in the report forms and analyzed. The ethics committee will then evaluate whether the trial should be suspended.

### Sample size

Due to the particularity of the neuroimaging mechanism, the clinical sample calculation method is not applicable. According to fMRI sample size research, each group should include at least 6–12 patients (40), while the statistical data of 20 subjects resembles that of large sample data for 130 subjects (41). Considering the uncertain factors in this study, including the loss or elimination of subjects and unavailable data due to head movements, we included 50 patients and 25 matched healthy persons in this study.

### Statistical analysis

#### Clinical data analysis

All baseline and clinical response index data will be analyzed using the SPSS software (version 22.0). Measurement data will be expressed as mean ± SD. Following the normality test, an independent samples *t*-test will be used if the criteria of normal distribution and homogeneity of variance are met. Otherwise, a non-parametric test (Mann–Whitney U-test) will be used. Count data will be analyzed by the two-sided χ^2^ test. A *p* < 0.05 will be considered significant.

#### fMRI data analysis

##### Neuroimaging data preprocessing

The DICOM software (version 1.3.5) will be used to convert the BOLD fMRI data into an analyzable NIFTI file. Preprocessing will be based on the MATLAB 2013b software platform with DPARSF V1.0 and SPM12 software. The preprocessing steps include removing the first 10 time points, time-layer correction, head-motion correction, spatial standardization, smoothing, delinear trends, noise filtering, and regression covariates.

##### Region of interest

Our previous studies found that multiple “DMN” core brain regions are closely related to urticaria, dyssomnia, and mood disorders. Their MNI coordinates are as follows: orbitofrontal cortex (*X* = −45, *Y* = 24, *Z* = −12), superior frontal cortex (*X* = −15, *Y* = 33, *Z* = 54), precentral gyrus (*X* = 48, *Y* = 9, *Z* = 33), angular gyrus (*X* = 45, *Y* = −48, *Z* = 33), and hippocampal gyrus (*X* = −18, *Y* = −12, *Z* = −9). These five brain regions were selected as regions of interest (ROI) to complete the resting-state, dynamic functional connectivity, and microstate analyzes.

##### Resting-state functional connectivity analysis

Based on the MATLAB 2013 software platform, the SPM12 software package will be used to perform the statistical analysis of brain functional connectivity. The whole-brain functional connectivity algorithm will be used to calculate the average time-series of all voxels at seed points. Next, all average time-series will be calculated individually with the Pearson correlation coefficient or the whole-brain voxel time-series. The correlation coefficient between the voxel and seed point can be obtained for each whole-brain voxel and transformed into an approximate Gaussian distribution data value using the Fisher-Z transform. Brain regions with significant statistical relationships will be identified according to a specific threshold and will be considered to have a resting-state functional connection with the seed points.

##### Dynamic functional connection analysis

The Dynamic BC 2.4 toolkit will be employed to analyze the dynamic functional connectivity network, and the sliding window correlation (SWC) method to analyze the dFC characteristics. Based on previous experience, a window length of 30TR (60 s) and a sliding step size of 1TR will be applied to generate 200-time windows. For each window, the intravoxel time-series Pearson correlation of the ROI will be individually calculated. Subsequently, each subject will produce a sequence of sliding window correlation coefficients, which will be transformed to a normal distribution using Fisher’s z-conform. The variation in the correlation coefficient time-series will be represented by calculating the standard deviation of the *z*-value for each voxel to characterize the dFC variability. Time means, standard deviations, and coefficients of variation will be used to analyze the specific attributes of dFC.

##### Brain micro-states extraction technology

Based on the dynamic functional connectivity network extraction, the k-means clustering method will be used in the dynamic functional connectivity matrix by cluster analysis to obtain the microstates of dynamic functional connectivity. Next, we will study brain functional connectivity changes in these microstates. The K-means algorithm uses a simple iterative strategy to divide the dataset into K nonoverlapping clusters. First, we will obtain the whole-brain dynamic functional connection vectors of all participants via vectorization in the upper triangular matrix of the dynamic functional connection matrix. Then, all functional connection vectors with strong variability (>1 SD) will be selected as clustering samples. The average diameter of each clustering result will be calculated to obtain the optimal number of clusters. The results will then be transformed into matrix form by running the standard k-means algorithm with the optimal clustering number on each subject’s dynamic functional connection vector. After summarizing, all clustering medians for each group will be used to represent the results for each group, i.e., the “micro-state.” Second, the time-series characteristics of the dFC microstates will be analyzed. The specific indicators include the average residence time of the microstate, conversion time, and stability.

#### Microbiota analysis

Clinical skin and intestinal flora DNA will be analyzed using 16S rDNA amplicon sequencing to determine the microbial composition of the samples. 16S rRNA gene sequencing will be performed using Novogene (Beijing, China), according to the manufacturer’s instructions. Raw data obtained by sequencing will then be spliced and filtered to obtain clean data. The DADA2 method will be employed to reduce noise based on the effective data, and sequences with abundances <5 will be filtered out to obtain the final amplicon sequence variants (ASVs).

For the ASVs obtained, species annotation will be performed on representative sequences to obtain the corresponding species information and abundance distribution. Simultaneously, the abundance, alpha diversity calculation, Venn diagrams, and petaline graphs of ASVs will be analyzed to obtain species richness and evenness information. Additionally, multiple sequence alignment of ASVs will be carried out to construct a phylogenetic tree. Principal coordinate analysis (PCoA), principal component analysis (PCA), nonmetric multi-dimensional scaling (NMDS), other dimension reduction analysis, and sample phylogenetic tree display will be applied to explore the differences in community structure between CSU patients and healthy people, and between the acupuncture group and sham acupuncture group. To further explore the community structure differences among grouped samples, statistical analysis methods, including *t*-test, MetaStat, and LEfSe, will be employed to test the significance of species composition and community structure of grouped samples.

#### Correlation analysis

The interactions between brain functional connectivity, microbiota, and clinical indicators in patients will be analyzed using MATLAB (version R2015b), which will calculate the Spearman correlation. An effect size of *r* = 0.10 is considered small (explained by 1% variance), 0.30 is medium (explained by 9% variance), and 0.50 is large (explained by 25% variance). The *Z* test and Fisher’s r-to-z transformation will be used to evaluate the differences in correlation coefficients between the acupuncture and sham acupuncture groups. Statistical significance is set at *p* < 0.05. Cytoscape (v. 3.8.2) will be employed to construct a visual network of interactions between brain functional connectivity, microbiota, and clinical indicators.

#### Data management and quality control

The clinical data will be recorded in a case report form (CRF) by a trained researcher. Another researcher will confirm the accuracy and completeness of the recorded information and record the relevant data in a password-protected Excel file. All CRF data will be stored in a locked cabinet for at least 5 years. The Biomedical Ethics Review Committee of West China Hospital, Sichuan University will regularly review the progress of the study and monitor the research data to ensure authenticity and reliability.

## Discussion

We hypothesized that the brain network mechanism of acupuncture serves as the multi-dimensional regulator of the key “DMN” brain network in CSU. To test this hypothesis, we will employ BOLD fMRI as the primary research method, as well as rsFC with strong spatial characterization, dFC with high sensitivity to time-varying characteristics, and microstate extraction as the main analysis methods. In this way, we aim to explore the pathological features of brain networks in patients with CSU in three dimensions: time, space, and microstates.

Microstate extraction technology will be used to identify subtle changes in the deep layers of the dynamic brain network. The “microstate” is a transient stable state found in many dFC studies (42). Specifically, the brain rapidly evolves into another state after maintaining a particular state for 80–120 ms; different states reappear over time. This analysis method has high sensitivity and specificity and can capture hidden dynamic changes based on dFC analysis.

In addition to studying the brain network, we will evaluate changes in the microbiota. Specific relationships between gut microbiota and CSU have been proven. Similar to the intestine, many skin microbiota are related to pruritus diseases (43). However, the direct relationship between skin microbiota and CSU remains unclear. Therefore, in this study, we evaluated the gut and skin microbiota to explain the pathological characteristics of CSU from a microbiota perspective.

Given the complexity of CSU pathogenesis, it is not sufficient to explore its mechanism using a single peripheral or central mechanism. Moreover, due to the overall regulatory characteristics of acupuncture, it is difficult to systematically explain the acupuncture treatment mechanism of CSU from a single perspective. Nevertheless, gut microbiota is significantly associated with multiple brain network functional connections, including DMN (44). Hence, this study will exploit the key brain networks of CSU as a breakthrough point to explore the acupuncture treatment mechanism for CSU in the “brain-gut-skin” context. Compared to previous acupuncture central mechanism studies for CSU treatment that only focused on single brain regions, we will explore the mechanism using rsFC, dFC, and microstate extraction to evaluate brain function while combining microbiota and clinical symptom index correlation analyzes. Hence, this study is expected to provide scientific and objective visual evidence for the clinical application of acupuncture in CSU treatment and promote further development of basic research on CSU treatment.

This study has certain limitations. First, the acupuncturists cannot be blinded due to the particularity of the acupuncture procedure, which may cause bias. Second, because of the skin sensitivity of patients with CSU, the blunt needles used in the sham acupuncture group may stimulate the patients’ skin, resulting in unpredictable changes in the neuroendocrine network, which may affect the experimental results. Third, as a single-center study, the number of samples will be small, and the results may not be extrapolated owing to the single operator, research equipment, and inclusion of patients.

## Author contributions

XH: Formal analysis. JX: Formal analysis. YY: Data curation. XD: Supervision. LL: Data curation. NL: Resources. YL: Funding acquisition. SW: Conceptualization. LZ: Conceptualization, Funding acquisition.
